# Case report: A case of perineal prolapse of giant uterine fibroids complicated by multiple pulmonary embolisms and deep venous thrombosis

**DOI:** 10.3389/fonc.2024.1415928

**Published:** 2024-05-24

**Authors:** Shixiang Dong, Xin Sun, Fengsheng Yu, Wenjie Wang, Li Zhang, Yankui Wang, Xiao Yu

**Affiliations:** ^1^ Department of Gynecology, The Affiliated Hospital of Qingdao University, Qingdao, China; ^2^ Medical College, Qingdao University, Qingdao, China; ^3^ Department of Pathology, The Affiliated Hospital of Qingdao University, Qingdao, China

**Keywords:** giant uterine fibroids prolapse from vagina complicated infection, intravascular leiomyomatosis, pulmonary embolism, deep vein thrombosis, inferior vena cava filter

## Abstract

A 43-year-old woman with a history of uterine fibroids, anemia, and deep vein thrombosis presented with a chief symptom of prolapse of tumor from the perineum, complicated by infection. The case was further complicated by bilateral pulmonary multiple embolism, deep vein thrombosis, acute cardiac insufficiency, acute renal insufficiency, and shock. The patient was treated with preoperative placement of an inferior vena cava filter, open hysterectomy, and perioperative anticoagulation with low-molecular-weight heparin. She smoothly navigated the perioperative period and recovered completely.

## Case presentation

In December 2022, a 43-year-old woman was hospitalized with a chief complaint of having had uterine fibroids for four years and experiencing irregular menstruation for the past three months. She had a history of uterine fibroids for four years and had a history of blood transfusion because of anemia. The patient had a menstrual cycle of 30 days, menstrual period of 4 days, moderate menstrual flow, and no dysmenorrhea. She had an obstetric history of G3P1 and had never used oral short-acting contraceptives. Pelvic examination showed a smooth cervix and a 3-cm tumor protruding from the cervical canal, which was polypoid and irregular in shape. Pelvic and abdominal examination suggested a tumor comparable in size to a 5-month gestational uterus, with a clear boundary and no obvious tenderness. Pathological examination of cervical neoplasia showed endometrial polyps. Transvaginal ultrasound did not show a normal uterine structure but identified a 20.6 cm × 17.7 cm × 10.7 cm hypoechoic mass with an irregular shape and uneven echo in the pelvic and abdominal cavities, which protruded downward out of the cervical canal over an area of 2.6 cm × 2.0 cm. Around this mass, another hypoechoic mass measuring 18.3 cm × 14.3 cm × 8.3 cm was detected. Lower limb vascular ultrasound showed a left lower limb intermuscular venous thrombosis measuring approximately 4.1 cm in length, with a D-dimer level of 1340 ng/mL, suggesting a new thrombosis. The patient had not got the COVID-19 immunization. She was infected with COVID-19, got a fever, and a chest CT revealed lung inflammation; therefore, her operation was postponed. After discharge, the patient stopped oral anticoagulant therapy with rivaroxaban and did not seek further medical consultation.

On December 14, 2023, the patient returned to the outpatient clinic complaining of a vaginal prolapse tumor appearing two days prior. She explained that the tumor had prolapsed from her vagina after coughing and could not be repositioned, accompanied by slight vaginal bleeding. The patient described feeling weak, experiencing slight chest tightness and suffocation, considerable abdominal and lumbosacral pain. She had difficulty urinating but had no chest pain, or swelling and pain in her lower limbs. Physical examination showed that the abdomen was distended, with a palpable hard mass in the lower abdomen ranging about 25 cm. The upper edge of the mass reached 4 cm above the navel. Mild edema was observed in the left lower limb. Pelvic examination showed that the vaginal prolapse tumor measured approximately 8 cm in length; it was columnar in shape and obstructed the vaginal opening. The tumor was purple-red in color, extending upward along the vagina, and was associated with a significant amount of pale yellow vaginal fluid with an unpleasant odor. The cervix was not palpable. In the pelvic and abdominal cavity, tumors comparable in size to more than six months’ gestational uterus were palpable, but these had no tenderness.

The patient was admitted to the hospital for emergency treatment. The admission diagnosis included possible uterine fibroids, vaginal prolapse tumor complicated with infection, and a history of deep vein thrombosis. Transvaginal ultrasound revealed a solid hypoechoic mass in the pelvis and abdominal cavity, measuring at least 27 cm × 24 cm × 14 cm. The internal echo was uneven, showing multiple irregular cystic cavities, with a ring-shaped strong echo observed at the edge of the mass. The upper part of the mass reached under the xiphoid process, and the lower part protruded outside the vaginal opening. After admission, the vaginal prolapse tumor continued to grow, although its color became ruddy. The patient was treated with indwelling catheterization, and vaginal secretions were collected for bacterial culture. Empirical anti-infection treatment was initiated with an intravenous drip of cefoperazone sodium and sulbactam sodium at a dose of 3.0 g every 8 hours.

**Figure 1:**
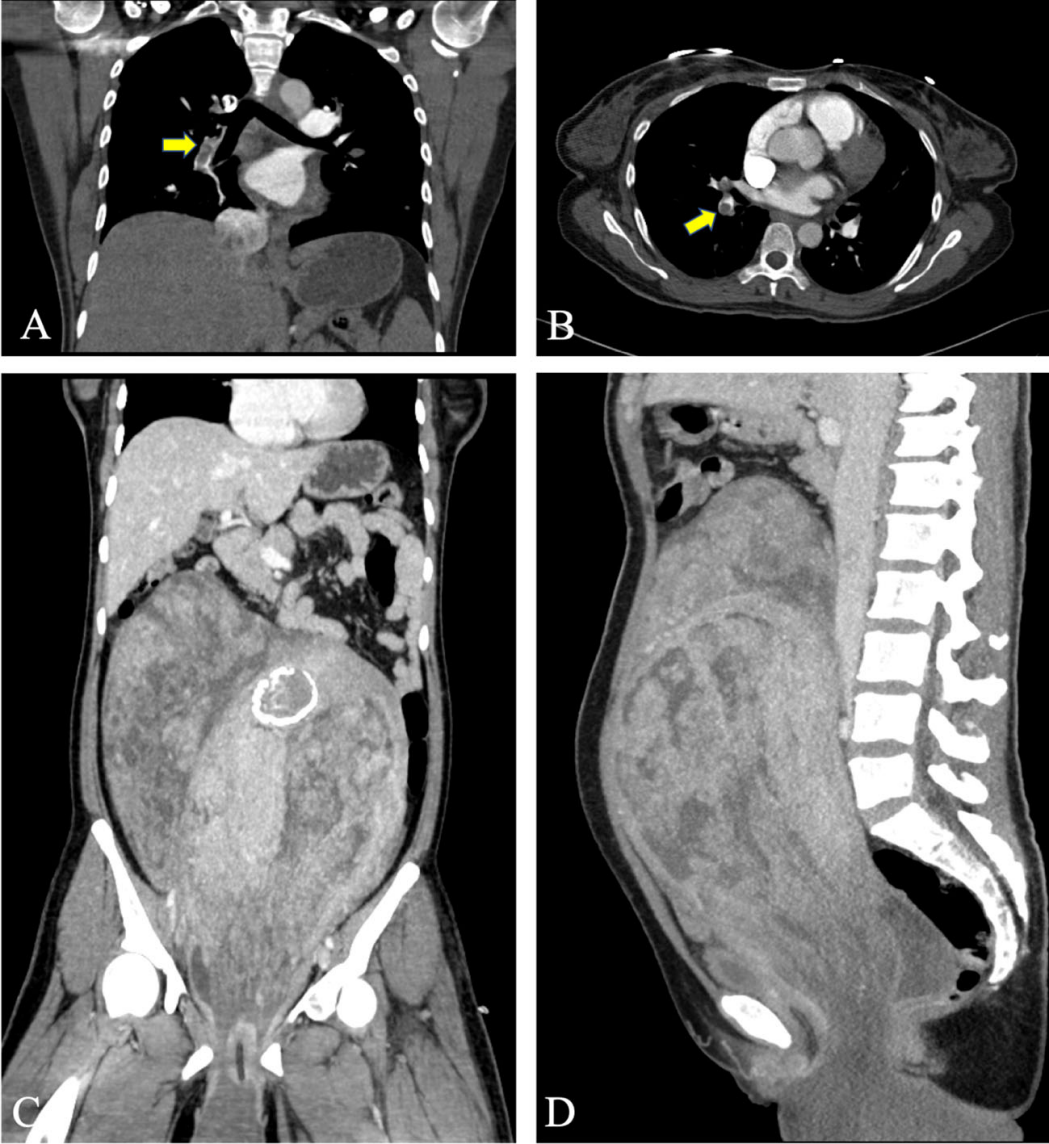
Preoperative CT evaluation of the patient. **(A, B)** CT angiography of the pulmonary artery shows multiple pulmonary emboli, and the arrow indicates the thrombus in the right pulmonary vessel. **(C, D)** A huge tumor can be seen in the pelvis and abdomen, with uneven density. Calcium density shadow can be seen, and the tumor protrudes downward out of the perineum.

Subsequent diagnostic tests revealed the following. D-dimer levels were considerably elevated at 14620 (reference range 0–500) ng/mL; prothrombin time and activated partial thromboplastin time of hemagglutination were normal; the white blood cell count was elevated at 15.25 × 10^9^/L (reference range 3.5–9.5 × 10^9^/L); hemoglobin levels were low at 84 (reference range 115–150) g/L; C-reactive protein levels were significantly increased at 61.55 (reference range 0–5) mg/L; procalcitonin levels were slightly elevated at 0.068 (reference range 0–0.05) ng/mL, and CA125 levels were elevated at 106 (reference range 0–47) U/mL. A lower-extremity vascular ultrasound revealed solid hypoechoic fillings in the left common femoral vein, popliteal vein, posterior tibial vein, peroneal vein, calf intermuscular vein, great saphenous vein, and small saphenous vein, leading to a diagnosis of deep venous thrombosis of the left lower extremity. Cardiac ultrasound showed a pulmonary artery pressure of 66 mmHg, indicating severe pulmonary hypertension, along with enlargement of the right atrium and right ventricle, reduced right heart function, and severe tricuspid regurgitation. CT angiography of the pulmonary artery showed multiple filling defects in various segments of both the left and right pulmonary arteries, including the left pulmonary artery, left upper pulmonary artery, left lower pulmonary artery, right pulmonary artery, right upper pulmonary artery, and right lower pulmonary artery, suggesting the presence of multiple thromboembolism in both pulmonary arteries, as shown in **Figure 1**. Fortunately, CT angiography of the inferior vena cava showed no thrombosis. During the examination, the patient experienced slight chest tightness, slight breath-holding symptoms, slight shortness of breath, and considerable abdominal pain. Measures were taken to manage her symptoms and potential complications; the patient was advised to elevate her left lower limb and lie in bed while restricting activities. Continuous ECG monitoring and oxygen inhalation were also initiated.

A myocardial enzyme test showed elevated levels of high-sensitivity troponin T at 0.333 (reference range 0–0.014) µg/L, suggesting acute myocardial injury. The patient had symptoms of oliguria, including decreased food intake, vaginal discharge, sweating, and only 50 mL of urine in 12 hours. Acute renal insufficiency was considered due to the patient’s inadequate intake, the urine volume not increasing substantially even after rehydration, and an increase in creatinine levels from 45.9 µmol/L to 89.6 (reference range 41.0–73.0) µmol/L. The patient’s left leg was obviously swollen, and she showed a progressive drop in blood pressure, with values between 85–90/45–60 mmHg. Blood oxygen saturation levels were maintained at 95%–99% with nasal tube oxygen inhalation at 3 L/min and dropped to 90%–95% in a non-oxygen inhalation state. The patient’s body temperature ranged from 36.0°C to 36.5°C, and her heart rate fluctuated between 85 and 95 beats/min. Blood gas analysis in an oxygen inhalation state revealed a pH of 7.39 (reference range 7.35–7.45) increased partial pressure of oxygen of 139 (reference range 83–106) mmHg, decreased partial pressure of carbon dioxide at 26 (reference range 35–45) mmHg, and elevated lactic acid levels at 4.6 (reference range 0.5–1.6) mmol/L. The patient had persistent hypotension and hyperlactatemia, indicating shock and circulatory failure. Consequently, she was transferred to the ICU for further treatment.

The patient was administered norepinephrine and other vasoactive drugs via an intravenous pump, and imipenem–cilastatin sodium at a dosage of 1 g every 8 hours for anti-inflammatory purposes. To improve oxygenation, red blood cell infusions were administered, and efforts were made to correct hypoproteinemia. The medical team carefully controlled the rate and volume of fluid infusion and provided appropriate diuretic treatment. During the treatment, a multidisciplinary team (MDT) consultation (imaging doctors, vascular surgeons, ICU doctors, anesthesiologists, cardiologists, respiratory doctors, and gynecologists) was arranged. The consensus from the consultation was that the pelvic and abdominal cavity tumors with infection were likely uterine tumors. Given the severity of the pain and indications for emergency surgery, the consultation highlighted the patient’s critical state, including the challenges posed by bilateral pulmonary multiple embolism; extensive deep venous thrombosis in the left lower limb; rapid disease progression; and severe comorbidities such as acute cardiac insufficiency, acute renal insufficiency, shock, circulatory failure, and hyperlactatemia. The risk of circulatory instability and respiratory and cardiac arrest during surgery was noted, considering the patient’s right ventricular dysfunction, which significantly increases the risk of mortality. Considering that the patient had symptoms of vaginal bleeding and a need for emergency surgery owing to the prolapsed huge tumor, low-molecular-weight heparin was selected for anticoagulation treatment. It was recommended that an inferior vena cava filter be placed before the operation to prevent the dislodgement of lower-extremity thrombi that could aggravate the pulmonary embolism. Therefore, the patient was administered low-molecular-weight heparin therapy, with instructions to stop the drug 12 hours before surgery, and efforts were intensified to improve the circulatory state of the patient.

After 12 hours of ICU support and treatment, the overall condition of the patient improved. Preoperative evaluation revealed that her body temperature had returned to normal, heart rate had stabilized at 80–90 beats/min, blood pressure had improved to 100–105/60–65 mmHg, with low-dose norepinephrine being administered intravenously, oxygen saturation was 100% with high-flow humidified oxygen therapy (FiO_2_ at 40%, flow rate 20 L/min), and the 12-hour urine volume had significantly increased to 1200 mL. Although the vaginal prolapse tumor had further increased in size, measuring about 25 cm × 20 cm × 20 cm, symptoms of abdominal compression had improved. However, the left lower limb remained obviously edematous. Laboratory examination showed that the D-dimer levels had decreased to 3960 ng/mL, white blood cell count had increased to 21.71 × 10^9^/L, hemoglobin level had dropped to 72 g/L, CRP level had decreased to 17.64 mg/L, PCT had increased to 0.170 ng/mL, N-terminal pro-B-type natriuretic peptide had increased significantly from 43.04 to 1733 (reference range 0–125) pg/mL, high-sensitivity troponin T had decreased to 0.075 µg/L, creatinine had decreased to 50 µmol/L, blood gas had returned to almost normal levels, and lactate had decreased to 1.4 nmol/L.

**Figure 2:**
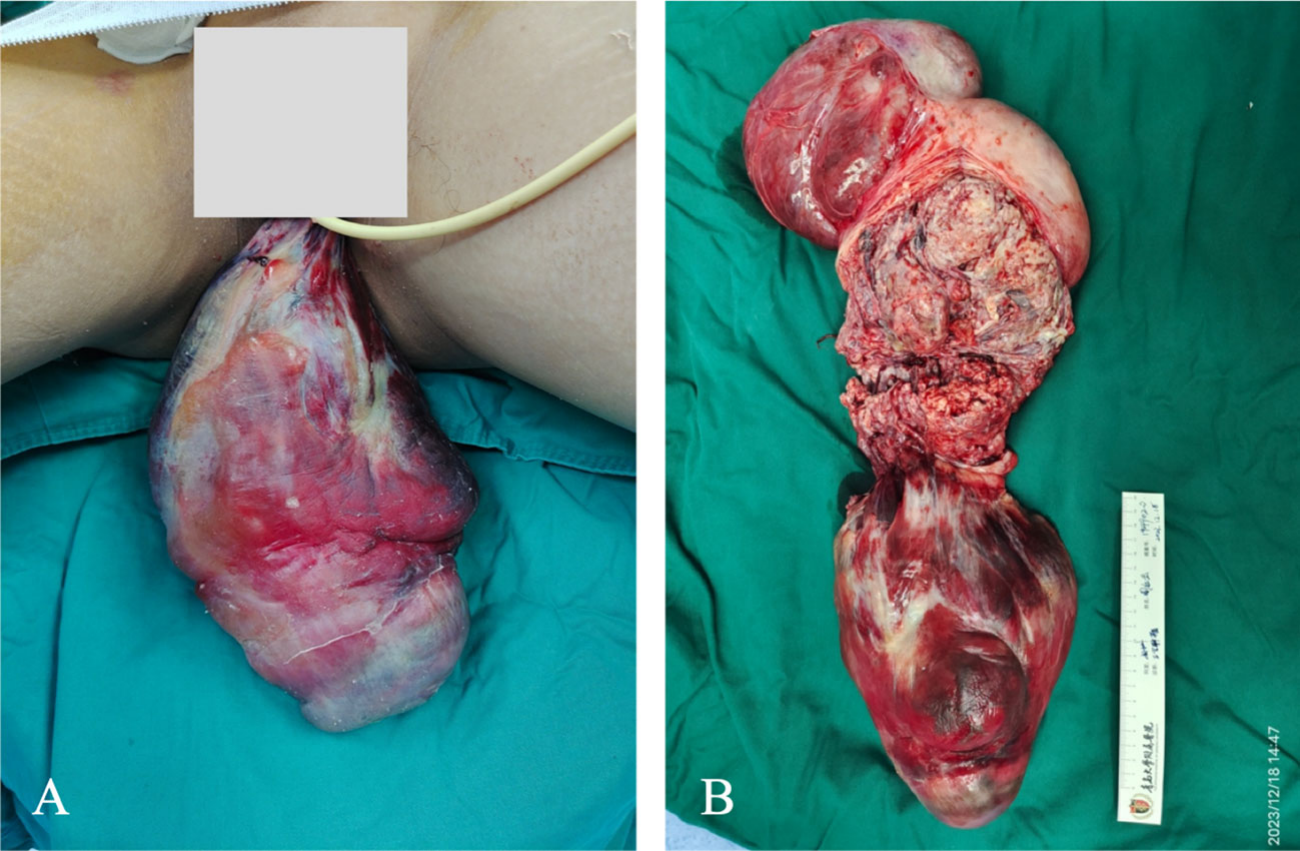
Intraoperative investigation of the patient. **(A)** The tumor prolapsed from the perineum measured about 25 cm × 20 cm × 20 cm. Parts of the tumor showed necrosis, while the majority was ruddy and had an unpleasant odor. **(B)** Dissection of the uterus.

**Figure 3:**
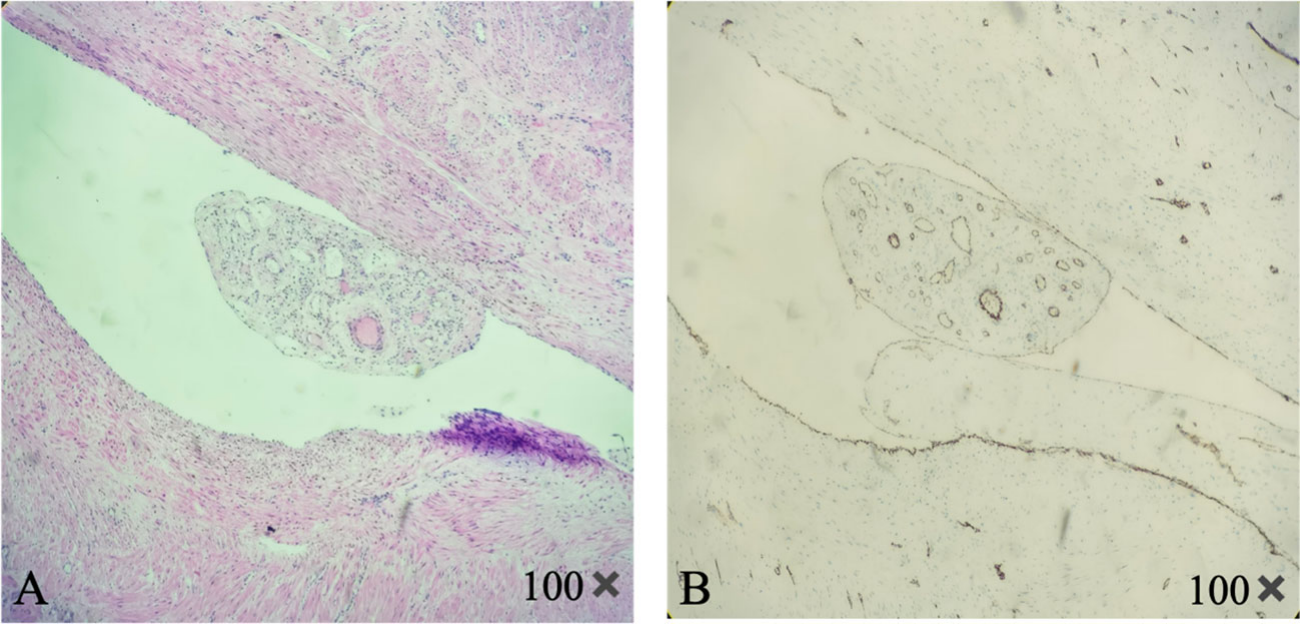
Pathology of the uterine tumor. The figure shows the lumen of the uterine vein, which contains tumor tissue derived from smooth muscle. Abundant blood vessels can be seen inside. **(A)** H&E staining **(B)** CD31 immunohistochemical staining shows tumor blood vessels.

The patient underwent emergency surgery on December 18, 2023, while minimizing traction on the tumor to avoid causing vagus nerve reflex that could aggravate the burden of circulation. The inferior vena cava filter was initially placed through the right femoral vein, positioning the filter at the L2–3 vertebral body level, and the blood flow to the inferior vena cava was unobstructed after the operation. The gynecological surgery was performed as an open procedure. During the operation, the uterine body was found to be comparable in size to a 5-month gestational uterus. A lobulated mass was palpable on the right side of the uterine body, measuring approximately 20 cm × 15 cm × 10 cm, protruding to the right broad ligament with a surface rich in blood vessels. The right accessory was pushed to the right rear of the uterine body by the right parametrial mass. There were abundant vascular bundles under the right infundibular ligament. The right ovary was deformed and elongated, and could not be retained. A decision to perform a hysterectomy, right adnexectomy, and left salpingectomy was made. The dissected uterus showed prolapsed tumor like submucosal fibroids, with edema and degenerative tissue changes, irregular boundaries in cotton-wool shape, and abundant blood vessels in the tumor, as shown in **Figure 2**. White, worm-like tissue growths were observed along the blood vessels, which could be pulled out, suggestive of endovascular leiomyomatosis. No tumor involvement was observed in the parametrial or mesosalpinx vessels of the resected uterine specimens. Intraoperative exploration of the parauterine and bilateral internal iliac vessels showed no obvious thickening or stiffening, and no intraluminal strip tumor bodies were palpated. Despite the surgical challenges, the operation proceeded smoothly with precise hemostasis. The intraoperative bleeding amounted to 100 mL, with an infusion volume of 3060 mL. The patient received four units of red blood cells and 290 mL of plasma. The excised uterine specimen weighed 5620 g. Initial rapid frozen section analysis suggested a diagnosis of leiomyoma. The intraoperative diagnosis included prolapsed uterine leiomyoma with infection, endovascular leiomyomatosis, bilateral pulmonary embolism, deep vein thrombosis, circulatory failure, acute cardiac dysfunction, acute renal dysfunction, severe pulmonary hypertension, severe tricuspid regurgitation, anemia, hypoproteinemia, and hyperlactatemia. Postoperative pathological examination revealed a smooth muscle-derived tumor with hemorrhage and infarction, with a huge tumor volume but without obvious atypia of tumor cells. Some areas showed degeneration and edema, and the specimen was rich in blood vessels. Tumor thrombus structures were observed in the focal large blood vessels of the uterine muscle wall, as shown in **Figure 3**.

The patient was treated in the ICU after the operation, where she continued to receive anti-infection and anticoagulation treatments. Anticoagulation therapy was resumed 12 hours after the operation with low-molecular-weight heparin, administered at a dose of 5000 IU subcutaneously every 12 hours. Efforts were also made to improve her anemia and provide nutritional support. After releasing the brake of the right lower extremity, the patient was treated with an intermittent pneumatic pressure device and antithrombotic pressure bandages. On postoperative day 1, ventilator support was discontinued, and vasoactive drugs were gradually reduced. On postoperative day 3, the patient was transferred back to the gynecological ward. Her symptoms of chest tightness and suffocation had resolved, and the swelling in her left lower limb had gradually improved. A CT angiography of the pulmonary arteries conducted seven days postoperatively showed that the pulmonary embolism was only seen in some branches of the bilateral lower pulmonary arteries but had significantly reduced. On postoperative day 10, the patient was successfully discharged from the hospital. She was prescribed oral rivaroxaban anticoagulant therapy (15 mg bid).

One month after the operation, CT angiography of the pulmonary artery revealed no thrombus. Vascular ultrasound identified a strip-like hyperechoic area at the beginning of the left deep femoral vein, measuring approximately 0.19 cm in width, which was interpreted as an old thrombus. The patient’s D-dimer levels decreased to 200 ng/mL, and other test results were normal. The patient had no symptoms such as chest tightness, suffocation, or lower-limb edema. In light of her recovery and stabilization, the anticoagulation therapy with rivaroxaban was adjusted to a maintenance dose of 20 mg once daily. Pulmonary artery CT angiography and lower limb vascular ultrasonography were reexamined three months following the initial surgery, and the findings were in concordance. The patient had no bleeding events and a D-dimer reading of 280 ng/mL. After successfully removing the inferior vena cava filter on March 29th, she resumed taking 20 mg of rivaroxaban once per day. After a month, she was planned to lower the dosage and discontinue taking rivaroxaban. We will follow up with the patient closely.

## Discussion

Intravenous leiomyomatosis (IVL) is a rare smooth muscle tumor that arises from the growth of uterine fibroids into blood vessels or from the proliferation of leiomyoma tissue in blood vessel walls. It initially develops in the uterine blood vessels; however, the lesion can grow beyond the uterus, along the pelvic veins to the inferior vena cava, and even invade the heart to cause pulmonary embolism, which can be life-threatening in severe cases (1). Venous thromboembolism (VTE) includes deep venous thrombosis (DVT) and pulmonary embolism (PE). Perioperative venous thrombosis is one of the causes of serious complications and death in patients (2). For DVT, the 30-day mortality rate ranges from 2% to 5.5%, while for PE, it ranges from 3.9% to 33%. The mortality rate of surgical patients has increased by over six times due to the development of VTE (3). The dense network of female pelvic veins, combined with venous plexus traffic between organs, exacerbated by factors such as anesthesia, immobility due to surgery, estrogen, and preoperative fasting, creates a hypercoagulable state. In addition, the specific position required for pelvic surgery (head low and feet high) increases the risk of DVT formation and detachment. If the thrombus falls off, it could pass through the inferior vena cava and the right heart into the pulmonary arteries, causing pulmonary embolism, which could be fatal in severe cases. Studies have reported that the probability of VTE is fourfold higher in cases of uterine fibroids weighing more than 1000 g compared to small fibroids (4). Another research reported that the median uterine volume of women who developed VTE was 2715 mL (5).

The patient had been diagnosed with venous thrombosis in the left lower extremity in 2022. The absence of formal anticoagulant treatment aggravated her condition. The huge tumor in the pelvic and abdominal cavity compressed the pelvic vein and inferior vena cava, leading to reduced blood flow. The tumor, combined with the patient’s anemia, caused a hypercoagulable state. The left common iliac vein was located between the right common iliac vein and the lumbosacral vertebra, making it susceptible to compression, resulting in slow blood flow in the veins of the left lower extremity. Furthermore, the infection of the vaginal prolapse tumor and the release of inflammatory factors worsened the situation. The above factors accounted for the filling of the deep and superficial veins of the patient’s left lower extremity with thrombi. The dislodgement of these venous thrombi led to multiple pulmonary embolisms in both pulmonary arteries. In this case, the intravascular leiomyoma was confined to the uterus, and the DVT and PE were not considered to be caused by intravascular leiomyomatosis.

The patient had multiple emboli in both pulmonary arteries, but the symptoms were not obvious. There were no typical pulmonary embolism manifestations such as chest pain, hemoptysis, or dyspnea. This discrepancy between clinical symptoms and diagnostic findings made pulmonary embolism difficult to identify without specific examinations such as cardiac ultrasound and pulmonary artery CT angiography. The patient’s symptoms were not consistent with the results of the auxiliary examination. Considering that the patient’s pulmonary embolism could be a combination of chronic and acute episodes, her cardiopulmonary function was in the compensatory stage at the time of admission. As her condition progressed, the mechanical obstruction caused by the thrombus and the pulmonary artery contraction caused by hypoxia led to severe pulmonary hypertension. This increased the afterload on the right ventricle, causing it to enlarge and leading to acute right heart dysfunction. Due to the limitation of pericardial volume, the left ventricular diastolic volume further reduced, and combined with pulmonary circulation disorder, reduced left cardiac output, causing acute renal dysfunction (prerenal). A large amount of fluid was retained in the tissue space, leading to significant edema in the lower limbs, decreasing venous return and aggravating circulatory and shock symptoms.

The expert recommends that surgical management of large uterine fibroids is contraindicated for a minimum of 4 weeks following diagnosis of acute DVT, and ideally delayed for 3 months to allow for thrombus stabilization (6). However, our patient’s situation was complex and was changing rapidly. The treatment of giant uterine fibroids prolapse complicated with DVT and PE was challenging and conflicting. There were two main goals of treatment, one was to stabilize and treat DVT and PE, and the other was to eliminate the predisposing factors, the tumor (7). According to the literature, there have been cases of giant uterine fibroids complicated by DVT and PE treated successfully; however, the majority of these patients received gynecologic surgery once the pulmonary embolism situation had stabilized (8–15), as shown in **Table 1**. None of these cases guided the emergency treatment of vaginal prolapse with a large tumor and infection. In order to assess the potential risks of various forms of therapy and the anticipated benefits for the patient, we arranged multidisciplinary discussions. We then discussed the treatment plan with the patient and ultimately made treatment decisions. The patient experienced a massive prolapse of uterine fibroid that was deteriorated by bleeding and infection, bilateral numerous emboli, and widespread thrombosis of the left lower extremity’s deep and superficial veins. Therefore, thrombolytic therapy, interventional therapy, or pulmonary thrombectomy were all restricted. Performing pelvic surgery in the state of acute pulmonary embolism was associated with a high risk and mortality rate. From the perspective of medical treatment of pulmonary embolism, low molecular weight heparin anticoagulation for at least 2 weeks was recommended before gynecological surgery, the patient has already developed circulatory failure, so timely surgical treatment was implemented. Relieving strain on pelvic and lower leg blood vessels, improving circulation significantly, and removing the restrictions of medication-assisted anticoagulant therapy could only be achieved by surgical intervention (16). Surgery should be performed after a short-term of preoperative anticoagulation, but anticoagulant therapy brings challenges to surgical treatment and increases the risk of bleeding before, during and after surgery. Furthermore, when pulmonary embolism was combined with circulatory insufficiency, the risk of anesthesia increased significantly. During patient transportation and surgery, the movement and compression of tumors protruding from the external genitalia and large tumors in the pelvic cavity may increase the risk of thrombosis and thrombus detachment, exacerbating pulmonary embolism. The key issue during the perioperative period was how to avoid new pulmonary embolism. Fortunately, the patient had no thrombus in the inferior vena cava. It was very necessary to place an inferior vena cava filter through interventional surgery before gynecological surgery to prevent further shedding of lower limb thrombus. Given that the patient is 43 years old and had no fertility requirements, open hysterectomy was chosen over uterine fibroid resection to minimize intraoperative bleeding, shorten the surgical time, and prevent recurrence of fibroids. After intraoperative wound hemostasis was verified, an abdominal drainage tube with a large diameter was inserted to get prepared to receive postoperative anticoagulant therapy. Under the guidance of an MDT consultation, a strategy was adopted that involved short-term active anticoagulation before surgery, rapid improvement of the circulation state of the patient, and placement of an inferior vena cava filter before the operation to prevent further dislodgement of the lower limb thrombus. An open hysterectomy was performed, along with careful bleeding management, and postoperative care in the ICU centered on stabilizing circulation, maintaining low-molecular-weight heparin anticoagulation, and administering anti-infection treatments. The patient smoothly navigated the perioperative period. One month following the procedure, a pulmonary artery CT angiography showed no thrombus and just a small amount of residual thrombus in the lower leg veins. The patient’s D-dimer levels went back to normal, and she denied any discomfort, which suggests a full recovery. Three months following surgery, the inferior vena cava filter was successfully removed.

**Table 1:** The summary of literature review.

Author	No. of cases	age	Diagnosis	Etiology	Implemented therapies	Weight of resected uterus (g)	Size of uterus (cm)
Satti MA et al.2016 (8)	3	47 60 41	DVT(L)+PE DVT(L+R)+PE PE	pelvic vein compression pelvic vein compression pelvic vein compression +blood transfusion	heparin+warfarin; IVC heparin+warfarin; IVC; hysterectomy massive PE; expired before surgery	—— 3010 ——	22×8.8×14 23.5×14×21.2 18×13×14.9
Cărbunaru A et al.2016 (9)	1	40	DVT(L)+inferior vena cava thrombus	pelvic vein compression	low-weight heparin; IVC; hysterectomy(2 weeks after IVC)	4500	25×21×20
Fernandes FL et al.2014 (10)	1	29	DVT(R)+PE	pelvic vein compression	low-weight heparin+warfarin; hysterectomy (4 months after thrombolytic therapy)	——	largest myoma 10×8×7
Khademvatani K et al.2014 (11)	1	42	DVT(L)+PE	pelvic vein compression+ medroxyprogesterone acetate	streptokinase+warfarin; myomectomy (3 months after thrombolytic therapy)	——	20-week size uterus
Kurakazu M et al.2012 (12)	1	40	PE	pelvic vein compression	heparin and tissue plasminogen activator under PCPS treatment; hysterectomy (12 days after thrombolytic therapy)	2700	22×12×11
Nishikawa H et al.2000 (13)	1	51	DVT(L+R)+PE	pelvic vein compression	heparin+warfarin; IVC; hysterectomy (7 days after IVC)	——	——
Bonito M et al.2007 (14)	1	49	DVT(L)+PE	pelvic vein compression	heparin; IVC; hysterectomy	5000	——
Unosawa S et al.2009 (15)	1	53	DVT+PE	pelvic vein compression +extreme obesity+ myomatous erythrocytosis syndrome	pulmonary thrombectomy; IVC; hysterectomy	2714	——

DVT, deep venous thrombosis; PE, pulmonary embolism; L, left; R, right; PCPS, percutaneous cardiopulmonary support; IVC, inferior vena cava filter.

This patient was a middle-aged woman with a huge uterine fibroid prolapsed from the vagina and complicated with infection, along with the presence of multiple pulmonary emboli and lower limb venous thrombosis. This case highlights the urgent need for early surgical intervention in patients with uterine fibroids when there are clear indications for such treatment. Moreover, it brings to light the importance of distinguishing IVL from more typical presentations of uterine fibroids. Gynecologists should pay attention to the early detection and management of potential complications associated with giant uterine fibroids, such as lower-extremity venous thrombosis, to reduce the incidence of pulmonary embolism and consequent mortality.

## Data availability statement

The original contributions presented in the study are included in the article/supplementary material. Further inquiries can be directed to the corresponding authors.

## Ethics statement

The studies involving humans were approved by the Ethics Committee of the Qingdao University Affiliated Hospital. The studies were conducted in accordance with the local legislation and institutional requirements. The participants provided their written informed consent to participate in this study. Written informed consent was obtained from the individual(s) for the publication of any potentially identifiable images or data included in this article.

## Author contributions

SD: Writing – original draft, Methodology, Data curation. XS: Writing – original draft, Data curation. FY: Writing – original draft. WW: Writing – original draft, Data curation. LZ: Writing – original draft, Data curation. YW: Writing – review & editing, Supervision. XY: Writing – review & editing, Supervision, Data curation.
